# Editorial: Emerging Translational Opportunities in Comparative Oncology with Companion Canine Cancers

**DOI:** 10.3389/fonc.2020.00270

**Published:** 2020-02-28

**Authors:** Mark W. Dewhirst, Rodney L. Page

**Affiliations:** ^1^Department of Radiation Oncology, Duke University, Durham, NC, United States; ^2^Colorado State University, Fort Collins, CO, United States

**Keywords:** comparative oncology, canine, cancer biology, cancer therapy, cancer diagnostic

The accelerated pace of discoveries in cancer biology is due, in large measure, to the engagement of an increasingly wide matrix of scientific disciplines focused on improved understanding and treatment of cancer. The intersection between physical sciences such as engineering, chemistry, biophysics, and mathematics with the traditional disciplines of biochemistry, cell biology, immunology, and genetics have created new opportunities to better define functional aberrations in the cancer process and explore novel concepts for prevention and management. This expanded understanding of the complexity inherent in cancer development and progression has occurred in parallel with the fundamental growth in technology that has accelerated resolution of the genetic, structural, and functional differences between normal, preneoplastic, and neoplastic conditions.

Vital to the application of new discoveries into clinical practice has been the vigorous development of preclinical systems for proof-of-concept studies, safety determination, and gauging potential efficacy. A vast number of genetically engineered laboratory animal models of human cancer are now available to resolve the importance of selective or aggregate alterations in the genetic code on critical biological functions. Although vital to preliminary confirmation of cancer discoveries, rodent models of cancer cannot fully recapitulate the complexity of driver mutations or tumor-host microenviroments seen in human cancer. Further, the presence of co-morbidities that occur in nearly all human patients contribute to tumor progression, treatment resistance, and normal tissue toxicities that cannot be readily modeled in rodents.

Unique similarities and differences in incidence, origin, development of cancer, and existence of co-morbidities between companion animals and humans makes studies in pet dogs directly applicable to people. To date, however, the value of canine cancer has been an underappreciated and incompletely developed scientific resource. In response to this gap, the veterinary profession established important components of a coordinated canine cancer research effort. Importantly, these efforts have been led by the Comparative Oncology Program at the National Cancer Institute (NCI Comparative Oncology Program). In 2015 the Institute of Medicine conducted a workshop to identify knowledge gaps and policy needs for integration of data obtained from clinical trials in companion animals for human drug development (Workshop Summary).

Substantial federal and non-profit research foundation resources have recently been committed to expand the scientific tools needed to better understand canine cancers and to coordinate multicenter clinical studies to expedite clinical cancer control strategies for humans and companion animals. Additional resources are needed to support identification of basic biological, immunological, and genetic drivers of neoplasia in companion animals, enhancements infrastructure and core resources for clinical trial management as well as enabling opportunities for proof of concept studies for industry. Once accomplished, these investments will permit more robust integration of canine comparative oncology with other cancer research resources.

This collection of review articles describes the current status of information relevant to comparative oncology in a variety of basic, preclinical and clinical categories. The goals of this curated collection are:

- To elucidate basic and pre-clinical research which has described how spontaneously arising cancers in dogs parallel the biologic complexity of human disease. Collection citations include—Langsten et al., Hlavaty et al., and Brocca et al..
- Discuss similar, as well as different, mechanisms of cancer progression in naturally occurring cancers of humans and companion dogs which are common across relevant histologies. For example, Interspecies translation of therapeutic targets for invasion, metastasis and drug resistance may support broadly applicable strategies due to the authentic mechanisms which are operative in both species. Collection citations include—Avery, Rao et al., Knapp et al., Miller et al., and Fan et al..
- Create awareness of the existing resources and capabilities available to produce and access high-value information obtainable in companion animal clinical cancer research—potentially improving the predictability of translational research. Collection citations include—Vail et al., Thamm, Dow, Nolan et al., and Etienne et al..
- Promote an increased level of collaboration between human and veterinary oncologists from both industry and academia in particular to narrow the gaps in awareness, understanding, and utilization that exist regarding companion animal data.

We believe that this collection of reviews and scientific manuscripts creates a representative cross-section of the discipline of comparative oncology in 2020 and, in aggregate, builds a roadmap for implementation of comparative oncology. The ability to fully integrate the biologic, genetic, and immunologic determinants of canine cancers into the broad landscape of cancer research is dependent on generation of a thorough portfolio of technological tools, clinical infrastructure and data repositories. Access to novel drugs and devices is then needed to demonstrate the business value of comparative oncology. Drug discovery case studies that demonstrate such value are accumulating, albeit slowly. Proper stimulation will generate additional interest, growth, and progress in cancer research for the benefit of all species.

## Author Contributions

MD and RP contributed equally to the review and editorial process for this collection.

### Conflict of Interest

The authors declare that the research was conducted in the absence of any commercial or financial relationships that could be construed as a potential conflict of interest.

